# Skin Mast Cell-Driven Ceramides Drive Early Apoptosis in Pre-Symptomatic Eczema in Mice

**DOI:** 10.3390/ijms22157851

**Published:** 2021-07-22

**Authors:** Piper A. Robida, Alena P. Chumanevich, Alexa Orr Gandy, John W. Fuseler, Prakash Nagarkatti, Mitzi Nagarkatti, Carole A. Oskeritzian

**Affiliations:** Department of Pathology, Microbiology and Immunology, University of South Carolina School of Medicine, 6439 Garners Ferry Road, Columbia, SC 29209, USA; parobida@nwosu.edu (P.A.R.); 2chumanevich@gmail.com (A.P.C.); Alexa.Gandy@uscmed.sc.edu (A.O.G.); John.Fuseler@uscmed.sc.edu (J.W.F.); PRAKASH@mailbox.sc.edu (P.N.); mitzi.nagarkatti@uscmed.sc.edu (M.N.)

**Keywords:** fluorescence microscopy, gene expression, immunology, inflammation, endoplasmic reticulum

## Abstract

Atopic dermatitis (AD or eczema) is the most common chronic inflammatory skin disorder worldwide. Ceramides (Cer) maintain skin barrier functions, which are disrupted in lesional skin of AD patients. However, Cer status during the pre-lesional phase of AD is not well defined. Using a variation of human AD-like preclinical model consisting of a 7-day topical exposure to ovalbumin (OVA), or control, we observed elevation of Cer C16 and C24. Skin mRNA quantification of enzymes involved in Cer metabolism [Cer synthases (CerS) and ceramidases (Asah1/Asah2)], which revealed augmented CerS 4, 5 and 6 and Asah1. Given the overall pro-apoptotic nature of Cer, local apoptosis was assessed, then quantified using novel morphometric measurements of cleaved caspase (Casp)-3-restricted immunofluorescence signal in skin samples. Apoptosis was induced in response to OVA. Because apoptosis may occur downstream of endoplasmic reticulum (ER) stress, we measured markers of ER stress-induced apoptosis and found elevated skin-associated CHOP protein upon OVA treatment. We previously substantiated the importance of mast cells (MC) in initiating early skin inflammation. OVA-induced Cer increase and local apoptosis were prevented in MC-deficient mice; however, they were restored following MC reconstitution. We propose that the MC/Cer axis is an essential pathogenic feature of pre-lesional AD, whose targeting may prevent disease development.

## 1. Introduction

Eczema, also known as atopic dermatitis (AD), is one of the most common chronic diseases, featuring severe itch and skin lesions. AD affects one-fifth of individuals residing in developed countries [1]. It is well established that a disrupted skin barrier plays an integral role in AD etiology. The stratum corneum (SC) is the outermost layer of the epidermis that prevents water loss and protects underlying tissues from external factors including allergens and pathogens. It comprises a lipid matrix of cholesterol, free fatty acids and ceramides (Cer) [2,3,4,5].

Cer are the most abundant SC lipids and play a critical role in maintaining homeostatic skin functions. Composed of a sphingoid base linked to a fatty acid chain, Cer can be produced by three distinct pathways: the de novo synthesis pathway requires serine palmitoyl transferase and ceramide synthases (CerS) to generate Cer, the salvage pathway involves degradation of complex sphingolipids to form Cer, and subsequently sphingosine and sphingosine-1-phosphate (S1P) by ceramidases and lastly the sphingomyelin pathway, which generates Cer from sphingomyelin and sphingomyelinases [6,7,8,9,10,11]. Cer regulate a variety of cellular responses, including cell cycle arrest and apoptosis, with specific behaviors being attributed to fatty acid chain length. Cer are classified as short, long or very long chains, based on the number of carbons (C) they feature, C16–C24 being the most common chain lengths [7]. Recent studies have highlighted the importance of Cer chain length in skin barrier dysfunction, highlighting a decrease in very long chain C24 at the expense of long-chain C16 during the chronic phase of AD [4,12,13]. However, the role of Cer during the early phase of AD has not yet been explored.

In the current study, we investigated the pre-lesional Cer composition of skin using a variation of a human-AD like mouse model after one 7-day exposure to Ovalbumin (OVA) or saline (vehicle control) to study disease initiation, the full model requiring three OVA exposures to induce chronic AD and skin lesions [14,15]. We found that epicutaneous OVA treatment of mouse skin resulted in significantly increased total Cer levels compared to saline controls. In particular, the levels of C16 and C24 Cer species were increased in OVA-treated samples. Elevated Cer result from increased synthesis and/or decreased catabolism. Therefore, local mRNA expression of Cer synthase (CerS) and ceramidase (Asah1/Asah2) was evaluated. CerS 4, 5 and 6 but also Asah1 mRNA levels were statistically augmented following OVA exposure. Given the overall pro-apoptotic nature of Cer [16,17], in situ activation of caspase 3, the executioner caspase of apoptosis [18], was measured using a novel image analysis method we describe to quantify cleaved caspase-3 (Casp3) detection in treated skin sections, also confirmed by protein analysis. Since apoptosis has been linked to endoplasmic reticulum (ER) stress [19], skin samples were further interrogated for key molecules of ER stress-induced apoptosis.

Mast cells (MC), skin-resident cells located near blood vessels, are well-known key players in the initiation of atopic diseases and an established source of a variety of pro-inflammatory mediators secreted upon activation by many stimuli [15,20,21,22,23]. To substantiate the relevance of MC in skin Cer elevation and apoptosis, the AD-initiating model was repeated in MC-deficient *Kit ^Wsh/Wsh^* mice, as previously described [15]. The absence of MC prevented OVA-induced Cer increase and local apoptosis. Moreover, primary bone marrow-derived MC (BMMC) activated in vitro by exogenous S1P, which we established as a key in vivo MC stimulus in pre-symptomatic AD [15], exhibited elevation of Cer C16 and C24 species with corresponding increased gene expression of *CerS 4*, *5* and *6* and *Asah1*. We propose that the MC/Cer axis is an essential pathogenic enabler of pre-lesional AD. In line with new directions for AD management and prophylaxis [24], our findings strongly suggest that targeting this axis may prevent disease development.

## 2. Results

### 2.1. In a Model of Preclinical Atopic Dermatitis, a Single OVA Exposure Triggers an Increase in Skin Ceramides

**Figure 1:**
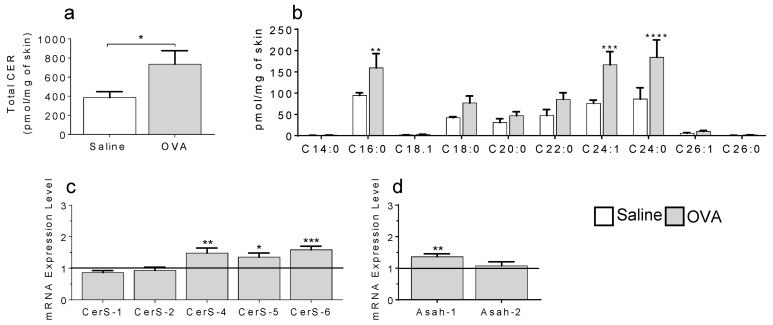
Skin exposure to OVA leads to increased ceramides in WT mice. OVA-induced skin total ceramide levels (**a**, * *p* = 0.0262), ceramide species (**b**, ** *p* = 0.0062; *** *p* = 0.0002; **** *p* < 0.0001), mRNA levels for ceramide synthases (CerS) (**c**, * *p* = 0.0153; ** *p* = 0.0088; *** *p* = 0.0001) and acid ceramidases (Asah) (**d**, ** *p* = 0.0013) compared to saline-treated mice. N = 6–8 mice per experimental group.

Cer, the most abundant skin lipids, are known to be decreased in the SC during chronic AD, resulting in increased dryness and exposure to external environment [25]. We have recently shown that localized changes occur very early in response to allergic stimuli [15,23]; thus, we sought to investigate the status of Cer at AD onset. Figure 1a (also see supplementary material) shows that a single 7-day epicutaneous OVA exposure nearly doubled Cer production compared to saline-treated controls.

Pertinent Cer species were highlighted upon skin Cer profiling, with Cer C16 and C24 species being most augmented (Figure 1b, supplementary material). To determine if the accumulated Cer were produced de novo, we explored the gene expression of Cer synthases (CerS). Figure 1c shows increased levels of CerS 4, 5 and 6 but not CerS 1 or 2 mRNA in OVA-treated mouse skin samples compared to saline, normalized to Gapdh (black line). We also observed increased gene expression for the acid ceramidase, Asah1, but not the neutral ceramidase Asah2 (Figure 1d). These results suggest that early accrual of Cer C16 and C24 in AD stemmed from both synthetic and catabolic pathways.

### 2.2. OVA Exposure Leads to Increased Detection of Cleaved Caspase-3

**Figure 2:**
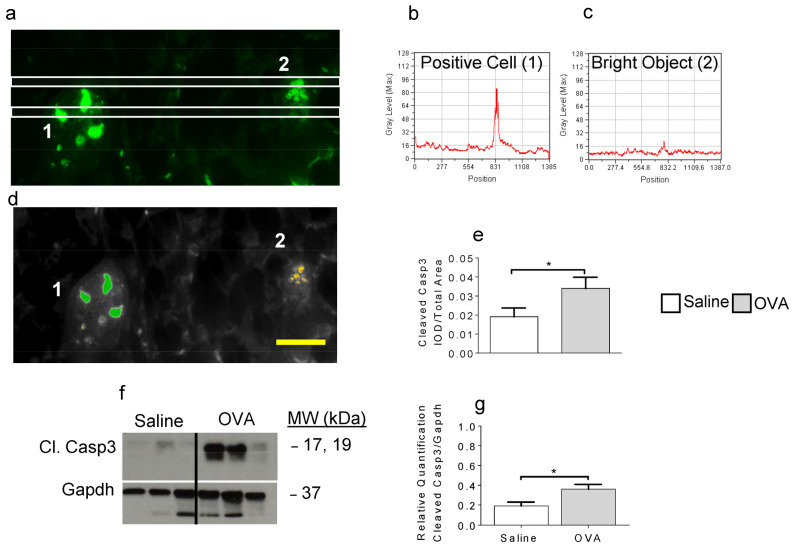
Skin-associated cleaved caspase-3 quantification following OVA exposure. Cleaved caspase-3 (Cl. Casp3) signal was detected in fluorescently labeled skin sections and quantitative parameters were developed as described in the methods section (**a**–**c**). Next, these morphometric parameters were utilized to threshold and measure positive signal (**d**1, green), distinct from nonspecific background signal (**d**2, orange). Quantification of Cl. Casp3 in skin samples (**e**, * *p* = 0.0486; n = 3–5 mice for a total of 40 images per group). Representative Western blot images for independent samples (**f**). Densitometry quantification of Cl. Casp3 proteins (**g**, * *p* = 0.035). Scale bar = 50 μm.

De novo production of C16 and C24 species has previously been linked to apoptosis [7]. Therefore, we next examined skin tissue sections for cleaved caspase-3 (Casp3). Figure 2a shows a representative image of an OVA-treated skin section fluorescently labeled with an anti-cleaved Casp3 Ab (see Methods).

Using a newly developed computer-assisted image analysis paradigm, line scans were applied to detect positive signal (Figure 2b) while eliminating non-specific and auto-fluorescence (Figure 2c). We have recently shown that morphometric parameters can be applied to quantify cellular changes in situ [26]. Similarly, we identified specific parameters for cleaved Casp3 fluorescence. In Figure 2d, we show that a positive signal can be thresholded and measured (in green) while simultaneously eliminating background staining (orange). By measuring the integrative optical density (IOD) over the total image area, we show a significant increase in cleaved Casp3 following OVA exposure compared to saline-treated animals (Figure 2e). The presence of cleaved Casp3 was further confirmed through western blot analysis (Figure 2f,g, supplementary material).

### 2.3. ER-Stress Contributes to Local Apoptosis Following OVA Exposure

**Figure 3:**
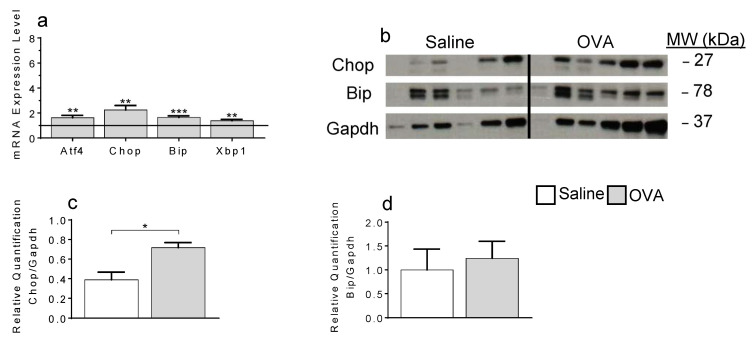
Endoplasmic reticulum stress-induced apoptosis-associated mRNA gene and protein expressions in skin samples. mRNA (**a**, ** *p* < 0.005; *** *p* = 0.0004) and protein (**b**–**d**, * *p* = 0.0173) expression of key ER-stress regulators. N = 5–7 mice per treatment group.

De novo synthesis of Cer, by CerS, occurs within the ER [27]. Increased Cer production can lead to ER stress, and ultimately apoptosis [28]. To determine if Cer-induced apoptosis occurred subsequently to ER stress, we examined skin tissues for stress-related markers [29]. Local mRNA levels of binding immunoglobulin protein (BiP), activating transcription factor 4 (Atf4), x-box binding protein 1 (Xbp-1) and C/EBP-homologous protein (Chop) were all augmented following OVA exposure (Figure 3a).

Furthermore, significantly more Chop, but not BiP, was detected by western blot in OVA-treated skins compared to saline alone (Figure 3b–d, supplementary material).

### 2.4. OVA-Induced Increase in Cer and the Resulting Apoptosis Do Not Occur in the Absence of Mast Cells

**Figure 4:**
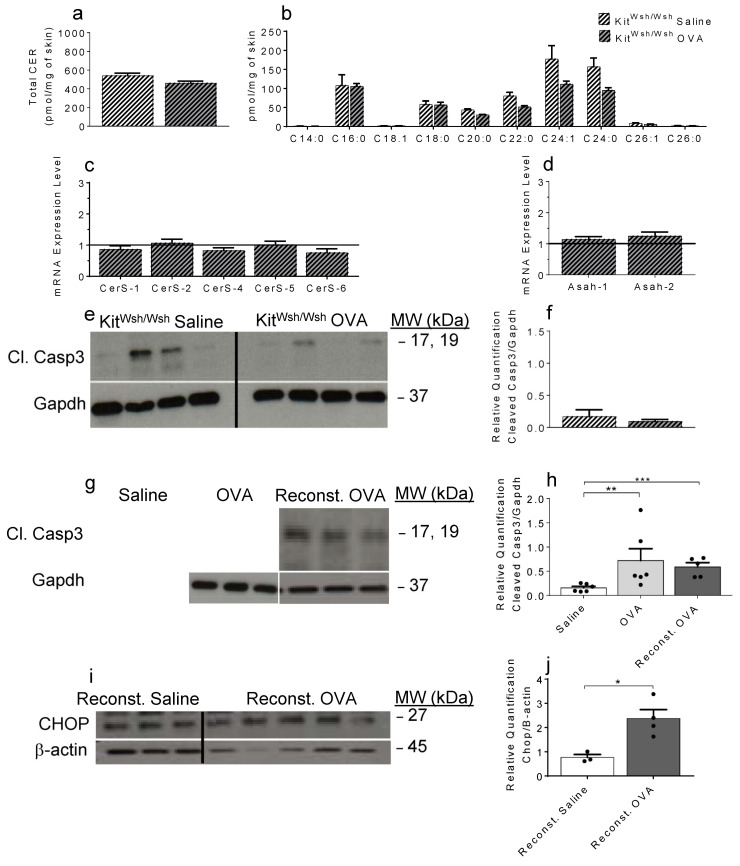
Ceramides, mRNA coding for genes involved in ceramide production and apoptosis in OVA-treated skin samples of *Kit^W-sh/W-sh^* MC-deficient mice. Skin-associated total Cer levels (**a**), Cer species (**b**), mRNA coding for CerS (**c**), and Asah 1 and 2 (**d**) in MC-deficient *Kit^W-sh/W-sh^* mouse skin samples. (**e**) Representative immunoblots for Cl. Casp3 of MC-deficient *Kit^W-sh/W-sh^* mouse skin samples (n = 4 mice per treatment) and densitometry quantification (**f**). Representative immunoblots for Cl. Casp3 of saline-treated WT, OVA-treated WT and OVA-treated MC-deficient *Kit^W-sh/W-sh^* mouse skin samples (**g**) and densitometry quantification (**h**, ** *p* = 0.0087; *** *p* = 0.0043; N = 5–6 mice per experimental group). Representative immunoblots for skin-associated CHOP of saline- and OVA-treated *Kit^W-sh/W-sh^* MC-deficient mice reconstituted with MC (Reconst.) (**i**) and densitometry quantification (**j**, * *p* = 0.0182; N = 3–4 mice per experimental group).

To substantiate MC’s contribution to skin Cer levels in vivo, we performed similar epicutaneous OVA exposure experiments in MC-deficient *Kit^W-sh/W-sh^* mice [30]. Here we show that in the absence of MC, OVA exposure does not lead to Cer accumulation (Figure 4a, supplementary material) or to elevation in C16 and C24 Cer species (Figure 4b, supplementary material) as we observed in MC-sufficient mice (Figure 1a,b).

Furthermore, OVA exposure does not contribute to changes in skin levels of any of the selected genes involved in Cer metabolism in skin samples of MC-deficient *Kit^W-sh/W-sh^* mice (Figure 4c,d), compared to WT mice (Figure 1c,d). In agreement with the absence of Cer elevation in *Kit^W-sh/W-sh^* mouse skins following OVA exposure, no change in cleaved Casp3 was measured upon OVA treatment (Figure 4e,f, supplementary material). Importantly, adoptive transfer of BMMC into the skin of MC-deficient mice restored OVA-induced apoptosis, as evidenced by increased levels of cleaved Casp3 (Figure 4g,h, supplementary material) and Chop (Figure 4i,j, supplementary material) proteins.

### 2.5. Bone Marrow-Derived MC, Activated In Vitro by Sphingosine-1-Phosphate, Recapitulate the Ceramide Profile Observed in OVA-Treated WT Mouse Skin Samples

**Figure 5:**
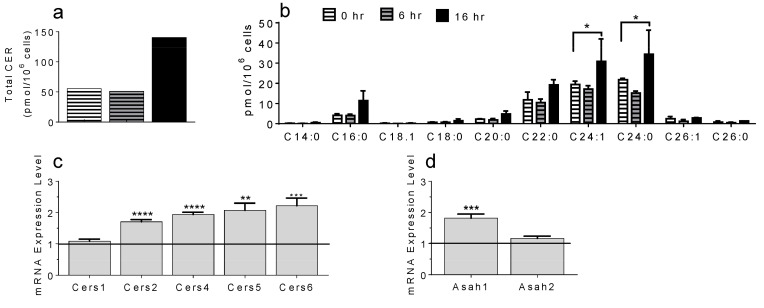
In vitro Cer generation in BMMC. Time-dependent production of total Cer (**a**) and Cer species (**b**, * *p* < 0.05) after activation by S1P. mRNA coding for CerS (**c**, ** *p* = 0.0024; *** *p* = 0.0017; **** *p* < 0.0001) and Asah (**d**, *** *p* = 0.0004) after 3 hours of S1P stimulation compared to untreated BMMC (black line in c and d). N = 4.

We recently highlighted the relevance of sphingosine-1-phosphate (S1P)-mediated activation of MC in vivo in an acute pulmonary model of anaphylaxis [23] and in eczema [15]. We next sought to determine whether exogenous S1P would trigger Cer production in MC. In Figure 5, bone marrow-derived MC (BMMC) were stimulated with S1P at different timepoints.

Total Cer were measured (Figure 5a, supplementary material) and Cer profiling was conducted (Figure 5b, supplementary material) after 0, 6 and 16 h of S1P activation. We found that total MC Cer levels were increased after 16 h of S1P stimulation, with Cer C16, C22 and C24 species being most prominent. Furthermore, S1P-mediated activation of BMMC resulted in a significant increase of mRNA coding for Cers 2, 4, 5 and 6 (Figure 5c) and ceramidase Asah 1 (Figure 5d) after 3 h of stimulation.

## 3. Discussion

Ceramides are well known to play a role in chronic AD, but their function at the early pre-symptomatic phase of AD is unclear. Here, we report that Cer accumulation can be observed in skin samples very early following a 7-day exposure to OVA, compared to saline controls. OVA exposure resulted in significant elevation of total Cer further classified as C16:0, C24:0 and C24:1. A recent study, conducted by YH Park et al., reported that increased C16:0 occurs at the expense of C24 species in a preclinical model of chronic AD, caused by down-regulation of the elongases, which create free-fatty acids longer than C16 [12]. However, it is important to note that (1) lipids were extracted from the SC layer only and (2) mice had manifestation of skin lesions, which do not present in our 7-day model. Furthermore, most of the studies focusing on Cer profiling utilize skin samples collected from patients already diagnosed with AD or from genetic mouse models of AD (recently reviewed in 24). By contrast, our study reports alterations of Cer profiles of mechanistic and pathogenic relevance using WT mice and epicutaneous exposure to antigen to better model the early onset of AD in non-predisposed humans or mice. Moreover, our results indicate a newly identified pathogenic function for ER stress-driven apoptosis at the inception of AD. While Cer are generally considered to be pro-apoptotic, it has been shown that C24 species can have protective effects [8]. As both pro-apoptotic C16 [31] and protective C24 species are significantly increased following one week exposure to OVA, it is tempting to speculate that pro-survival mechanisms are in effect to return affected skin tissues to homeostatic conditions. Accumulation of both C16 and C24 Cer has been observed within malignant tumors of head and neck squamous cell carcinoma and breast cancer patients compared with normal and/or benign tumor tissues [32,33], as well as in patients with uncontrolled asthma [34], suggesting that the balance between C16 and C24 species may control both apoptotic and survival pathways.

In addition to increased Cer levels, elevation of mRNA expression for Cer-generating enzymes was also observed. Cer synthases (CerS) 1–6 link specific subsets of fatty acyl-CoAs to a sphingoid base generating Cer with varying chain lengths, while ceramidases (acidic, neutral and alkaline) catalyze the cleavage of Cer. Here, we show increased expression of CerS 4, 5 and 6. It has previously been shown that CerS 5 and 6 are specific for long chain Cer, including C16. CerS 4, the least studied CerS, is considered to be a broad-spectrum CerS. Expression of CerS 4 has previously been linked to increased C24 Cer species [35]. Tirodkar et al. recently reported that elevation of CerS6 could mediate the transcriptional activation of Asah1, which may explain the increased detection of acid ceramidase we observed following OVA exposure [36].

In this study, we report that topical OVA exposure induces an increase in C16 and C24 Cer species compared to saline-exposed controls. Previous studies have linked de novo generation of C16 and C24 Cer to spontaneous neutrophil apoptosis [7] and tumor-induced dendritic cell apoptosis [37]. Similarly, we report that substantially increased C16 and C24 Cer is linked to apoptosis, as measured by skin-associated immunofluorescence signal upon OVA treatment. Apoptosis was confirmed through augmented quantification of cleaved Casp3 protein in OVA compared to saline-exposed skin samples by western blot. Thus, it appears that the pro-apoptotic effects of C16 surpass the protective effects of C24 at the initiation of AD.

When injury occurs, the ER stress response initiates survival pathways. The chaperone protein, BiP, dissociates from IREI, ATF6 and PERK, activating the unfolded protein response (UPR). If the UPR fails, the ER-stress apoptosis pathway initiates, involving CHOP and the caspase cascade [38]. Our results indicate that skin mRNA expression of BiP and transcription factors Atf4, Xbp1 and Chop were increased following OVA application, with confirmed elevation of CHOP protein, a key mediator of ER stress-induced apoptosis.

We have previously shown the importance of MC during early allergic lung inflammation [23] and during early-phase pre-symptomatic eczema by comparing lung and skin inflammation in MC-deficient *Kit^W-sh/W-sh^* mice and MC-reconstituted *Kit^W-sh/W-sh^* mice to WT, MC-sufficient mice of similar genetic background [15]. Thus, we sought to investigate MC’s contribution to the augmentation of Cer during early skin inflammation in similar models. In MC-deficient *Kit^W-sh/W-sh^* mice, OVA application did not trigger Cer production and the absence of Chop and cleaved Casp3 elevation supported lack of skin-associated apoptosis. Importantly, MC deficiency in this model could be corrected through skin MC reconstitution by adoptive transfer of WT BMMC, which restored the ability of skin exposure to OVA to induce apoptosis in levels comparable to MC-sufficient mice. These results further established a critical role of MC in the pathogenic dysregulation of skin, leading to the appearance of lesions in an overt diseased state. Our group discovered an important causative function for local elevation of skin S1P levels in triggering MC activation in pre-symptomatic eczema [15]. Similarly, we demonstrated that S1P activation of mouse BMMC in vitro stimulated Cer production pathways thus remarkably recapitulating the Cer profile observed in OVA-exposed WT mouse skin specimens. These findings further substantiated an essential role for MC in AD pathogenesis through MC contribution to the elevation not only of skin S1P levels [15] but also of skin Cer, identifying ER stress-induced apoptosis as an early event, eventually leading to skin lesions in AD. As current reports emphasize the need to develop new strategies to prevent the spread of skin lesions in AD patients, we propose that targeting S1P/MC interactions may represent a novel prophylactic approach to prevent Cer-mediated apoptosis at the onset of AD.

## 4. Materials and Methods

### 4.1. Atopic Dermatitis Model

AD was induced at 8 to 12 weeks in female C57Bl/6J mice as previously described [14,15]. Mice were purchased from Charles River NCI (Frederick, MD). In some experiments, MC-deficient *Kit^W-sh/W-sh^* (B6.Cg-*Kit^W-sh^*/HNihrJaeBsmGlliJ) mice and their corresponding WT controls were purchased from The Jackson Laboratory (Bar Harbor, ME). In some experiments, WT BMMC were transferred by intradermal injection of 4 × 10^6^ cells in 8 × 50-μL aliquots in two rows down the length of shaved back skin per 4-week-old female *Kit^W-sh/W-sh^* mouse, to locally reconstitute skin MC, exactly as described [30]. Nine weeks after intradermal adoptive transfer of WT BMMC, OVA or saline exposures of the back skin of MC-deficient mice locally reconstituted with MC were initiated. Skin tissues assessed for local repair of mast cell deficiency using methylene blue staining and WT control mice demonstrated proper reconstitution of dorsal skin MC [15,26,30]. Mice were randomized as described previously [15] and assigned to either OVA-treated or saline control experimental groups. Briefly, 100 µL of OVA solution (100 µg OVA (Sigma-Aldrich, St Louis, MO, USA) in 0.9% saline) or 0.9% saline only were pipetted on a 1 cm × 1 cm square gauze pad (patch) placed on the shaved and tape-striped upper-back skin area. Next, this area was occluded with a Tegaderm Transparent Dressing (3M HealthCare, St. Paul, MN, USA) using bandages. The patch was removed after seven days and skin samples were collected from euthanized mice.

### 4.2. Mass Spectrometric Measurements of Cer

Lipids were extracted from snap-frozen skin tissues and S1P-activated BMMC and Cer were measured by liquid chromatography-electrospray ionization-tandem mass spectrometry, as previously described [39] (LC-ESI-MS/MS; 4000 QTRAP; AB Sciex, Concord, ON, Canada). The quantification of Cer species was normalized to the weight of the skin sample or per 10^6^ BMMCs.

### 4.3. QRTPCR

Skin samples were collected, snap-frozen and stored at −80 °C until RNA extraction. The total RNA from the patched skin samples and S1P-activated BMMC cell pellets was isolated and purified with the miRNeasy kit (Qiagen, Valencia, CA, USA), following the manufacturer’s procedure. The iScript cDNA synthesis kit (Bio-Rad, Hercules, CA, USA) was used according to the manufacturer’s specifications to reverse transcribe cDNA. QRTPCR was performed on a CFX Connect (Bio-Rad) with SensiFAST™ SYBR No-ROX Kit (Bioline, Taunton, MA, USA). The primer sequences (Thermo Fisher Scientific) used for QRTPCR amplification can be found in Table 1. The QRTPCR conditions were as follows: the initial step at 95 °C for 5 min and cycles (*n* = 40) consisted of 10 s at 95 °C, followed by 1 min annealing at 55 °C and extension at 72 °C. All reactions were performed in duplicate. Data were analyzed with CFX Manager™ Software and their expression was normalized to saline-treated samples for in vivo experiments or 0 h S1P activation for in vitro experiments and were directly proportional to the amount of mRNA of the target gene relative to the amount of mRNA of the reference gene, i.e., glyceraldehyde 3-phosphate dehydrogenase (*Gapdh*) mRNA levels [15,40].

### 4.4. Immunofluorescence Quantification of Cleaved Caspase-3 in Skin Sections

Skin tissues collected from euthanized mice were fixed in 4% paraformaldehyde, paraffin-embedded, sectioned (4 μm thickness) and mounted on microscope slides. Tissues were subjected to dewaxing, rehydration, antigen retrieval, blocking and permeabilization prior to overnight incubation with anti-cleaved Casp3 antibody (CST, Danvers, MA, USA). Secondary AlexaFluor 488 donkey anti-rabbit IgG Ab (Jackson ImmunoResearch, West Grove, PA, USA) was used to visualize cleaved Casp-3. Images ((40×) magnification) were collected as TIFF files and analyzed. Morphometric analysis was performed using the MetaMorph^®^ 6.1 Image Analysis software (Molecular Devices, Sunnyvale, CA, USA) and our expertise in quantitative imaging [15,26], to measure cleaved Casp-3 *in situ*, as follows: we used the line intensity scan function as a graphical tool to measure the intensity of cleaved Casp-3 fluorescence signal present between two horizontal rows of pixels and to determine the average values over several adjacent pixel rows in 40× digital images of skin sections (Figure 2a). We established intensity scan profiles (Figure 2b,c), which display the graphs of scan position (*x*-axes) versus the fluorescence intensity converted into gray scale values (*y*-axes) for each pixel row lying between the two white horizontal scan lines traversing each digital image. Thus, the intensity/gray scale values corresponding to each horizontal position on the scan line were computed as the arithmetic mean of the intensity values of all of the pixels in the row. The average gray scale value for the pixels bounded by the scan lines was plotted along the major axis of positive signal using the line scan sub-routine of MetaMorph image analysis software. In sum, we used the line intensity scan function to generate graphs of pixel intensity values of cleaved Casp-3 measured at each position along a horizontal scan line through each digital image. Next, we related pixel brightness information collected by line scan to another quantitative morphometric parameter, the integrated optical density (IOD), measuring the amount or mass of cleaved Casp-3 contained in a digital image as previously reported [15,26]. The values of IOD were calculated directly from the integrated morphometry subroutine of MetaMorph 6.1 software. Using the software’s optical calipers, the measurements were refined by setting specific boundary conditions for total area and IOD for the acceptance of individual cell-associated signal (1 in Figure 2d) and to eliminate the contributions of any non-specific or background staining (2 in Figure 2d, [26]). The concept of IOD representing the mass or total amount of stained material in an image is well established (detailed in [26]). Finally, we calculated ratios of cleaved Casp-3 IOD over the total area, thus combining two measurements of the gray levels at the same spatial location in each image to quantify the total amount of cleaved Casp-3 in differentially treated skin specimens (Figure 2e).

### 4.5. Western Blots

Proteins were extracted from snap-frozen skin tissues and the Bradford protein assay was used to measure whole skin lysate protein concentrations. Equal amounts of proteins were electrophoretically separated on a 4–20% Mini-PROTEAN^®^TGX^TM^ gel (Bio-Rad, Hercules, CA, USA) followed by electrophoretic transfer to a polyvinylidene difluoride (PVDF) membrane. Membranes were immunoblotted with primary Ab (Cleaved Casp-3, Chop, BiP and Gapdh; CST, Danvers, MA, USA). Proteins were detected using a horseradish peroxidase (HRP)-linked secondary Ab, followed by incubation with an HRP substrate for ECL (ThermoFisher Scientific, Waltham, MA, United States) and captured on film. Results are shown for individual mice treated with saline or OVA. Signal quantitation was carried out with ImageJ software (National Institutes of Health, Bethesda, MD, USA). Integrated density numbers (in pixels) of cleaved caspase-3, Chop or BiP were normalized to Gapdh or β-actin loading controls, as indicated.

### 4.6. Culture and Activation of Bone Marrow-Derived Mast Cells

Mouse BMMC were generated from progenitors isolated from femurs and cultured in RPMI 1640 medium (Invitrogen Life Technologies, Carlsbad, CA, USA) containing 10% heat-inactivated FBS, 2 mM L-glutamine, 1% antibiotics, 1 mM sodium pyruvate, 1 mM HEPES (Biofluids, Rockville, MD, USA), 2 ng/mL recombinant mouse IL-3 and 20 ng/mL of recombinant mouse stem cell factor (SCF, PeproTech, Rocky Hill, NJ, USA), exactly as described [41]. After 4 weeks of culture, BMMC were more than 98% pure and fully matured. S1P (100 nM) or vehicle (PBS-4 mg/mL FA-free BSA) was used to stimulate BMMCs for Cer measurements and QRTPCR analyses.

### 4.7. Statistics

Data are expressed as means ± SEM (unless otherwise stated) and were analyzed by using the unpaired two-tailed Student *t* test, with Welch’s correction for samples of unequal variance (Prism 6; GraphPad Software, La Jolla, CA, USA). The significance for all statistical tests is shown in the figures and figure legends. The experiments were repeated at least three times in triplicates with consistent results. The in vivo experiments were repeated three times, and each experimental group consisted of five to six mice.

## Figures and Tables

**Table 1:** QRTPCR murine primer sequences.

Target	Forward Sequence	Reverse Sequence
Mu Gapdh	CAG AAG GGG CGG AGA TGA T	AGG CCG GTG CTG CTG AGT ATG TC
Mu Cers1	CCA CCA CAC ACA TCT TTC GG	GGA GCA GGT AAG CGC AGT AG
Mu Cers2	ATG CTC CAG ACC TTG TAT GAC T	CTG AGG CTT TGG CAT AGA CAC
MuCers4	TAC CCA CAT CAG ACC CTG AAT	TGA AGT CCT TGC GTT TGA CAT C
Mu Cers5	CGG GGA AAG GTG TCT AAG GAT	GTT CAT GCA GTT GGC ACC ATT
Mu Cers6	ATT CAA CGC TGG TTT CGA CAA	TTC AAG AAC CGG ACT CCG TAG
Mu Asah1	CAC CAG CGT TGA GGA TTT TAG T	TAC CAG GCA GCT TTT GAT CCA
Mu Asah2	GCA AAG CGA ACC TTC TCC AC	ACT GGT AAC AAA CAA GAG GGT GA
Mu Casp3	ATG GAG AAC AAC AAA ACC TCA GT	TTG CTC CCA TGT ATG GTC TTT AC
Mu Atf4	GGG TTC TGT CTT CCA CTC CA	AAG CAG CAG AGT CAG GCT TTC
Mu Chop	CCA CCA CAC CTG AAA GCA GAA	AGG TGA AAG GCA GGG ACT CA
Mu Bip	TTC AGC CAA TTA TCA GCA AAC TCT	TTT TCT GAT GTA TCC TCT TCA CCA GT
Mu Xbp1	GAA CCA GGA GTT AAG AAC ACG	AGG CAA CAG TGT CAG AGT CC

## Data Availability

Data supporting the reported results can be found in the Supplementary Materials.

## Supplementary Materials

Supplementary materials can be found at https://www.mdpi.com/article/10.3390/ijms22157851/s1. Mass spectrometry datasets are included for Figure 1, Figure 4a,b and Figure 5a,b. Original images of western blots are included for Figure 2f, Figure 3b and Figure 4e,g,i.
