# Supplementary material for: Skin Mast Cell-Driven Ceramides Drive Early Apoptosis in Pre-Symptomatic Eczema in Mice
Source: Int J Mol Sci. 2021 Jul 22;22(15):7851. doi: 10.3390/ijms22157851 (PMC8346072; doi:10.3390/ijms22157851)
Supplement: Supplementary file 1 [file ijms-22-07851-s001.zip › Original Images for Western blots.pdf]

Original Images for Western blots:

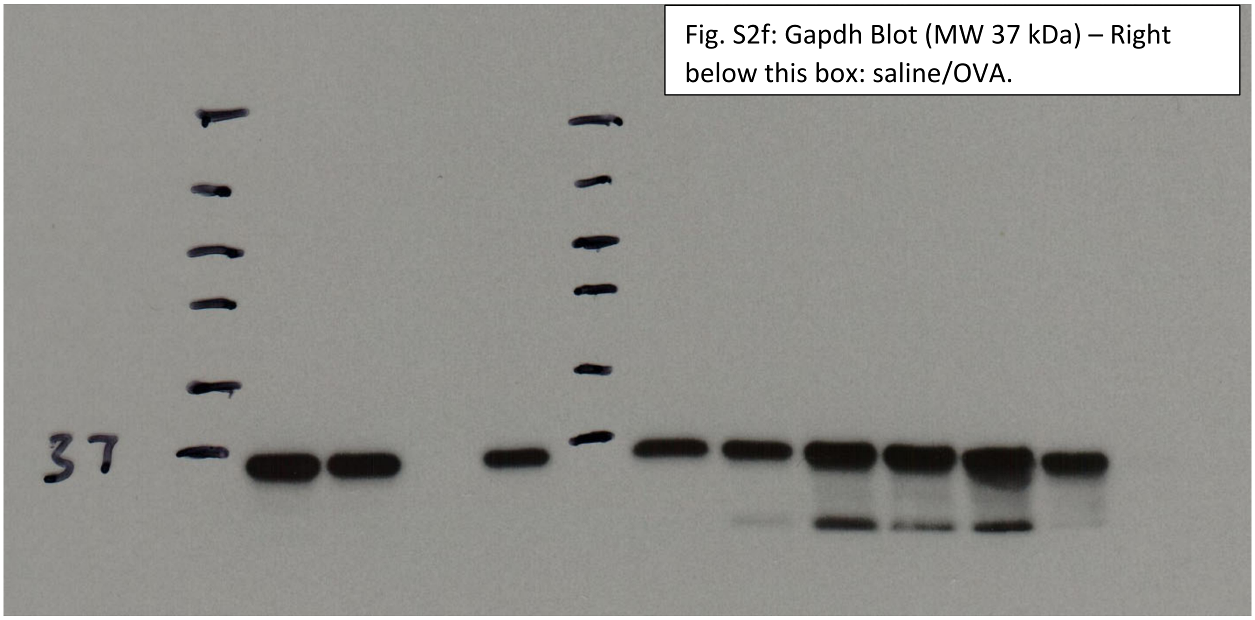

Fig. S2f: Gapdh Blot (MW 37 kDa) – Right below this box: saline/OVA.

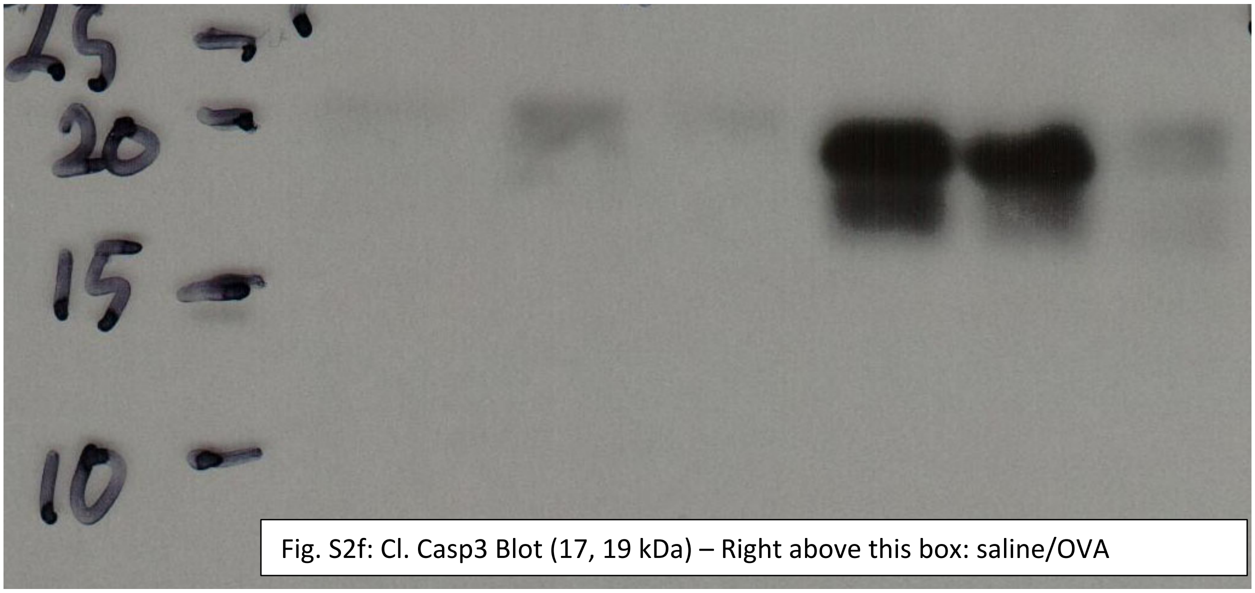

Fig. S2f: Cl. Casp3 Blot (17, 19 kDa) – Right above this box: saline/OVA

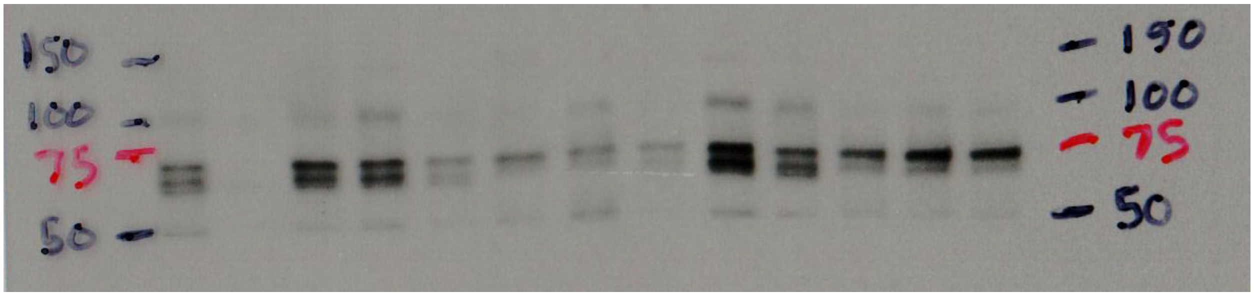

Fig S3b: Bip (78 kDa) – Above this box:saline/OVA

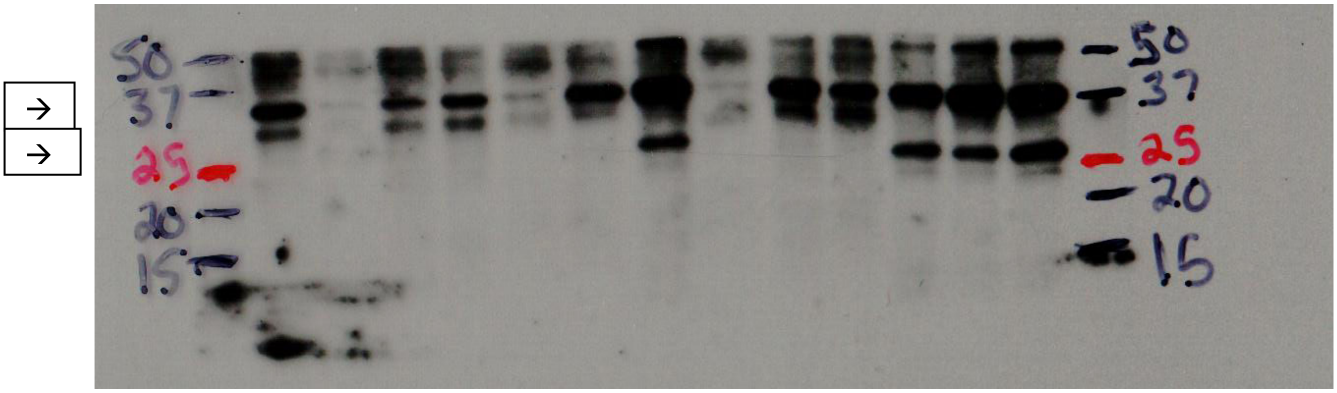

Fig S3b: Chop (27kDa) and GAPDH (37kDa)—Above this box:saline/OVA

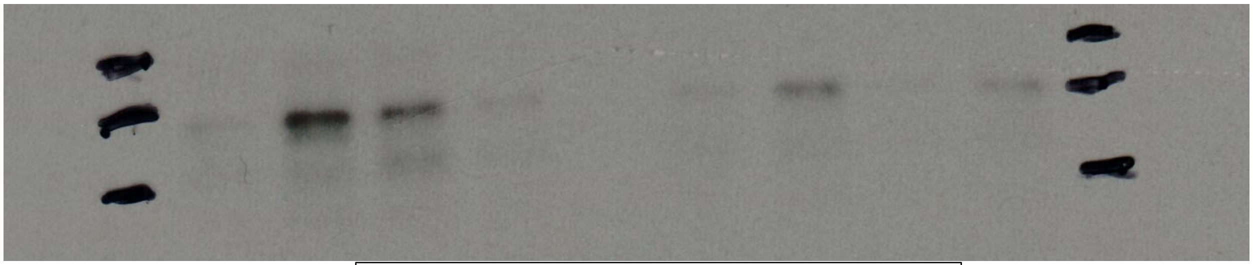

Fig. S4e: Cl. Casp3 (17,19kDa) – Saline/OVA

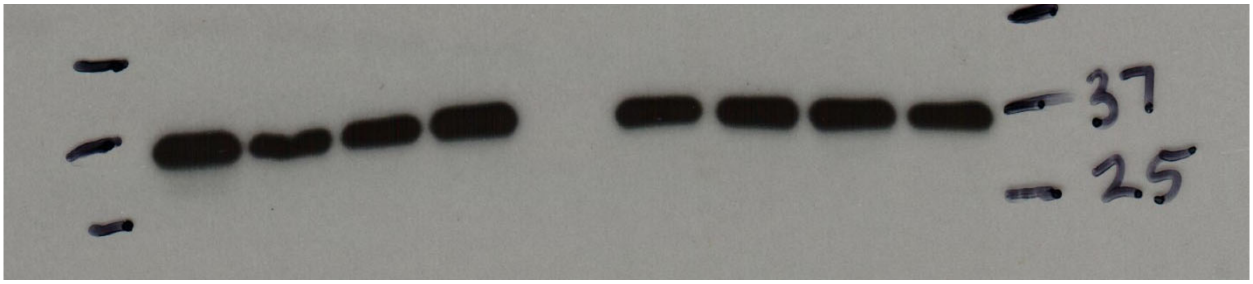

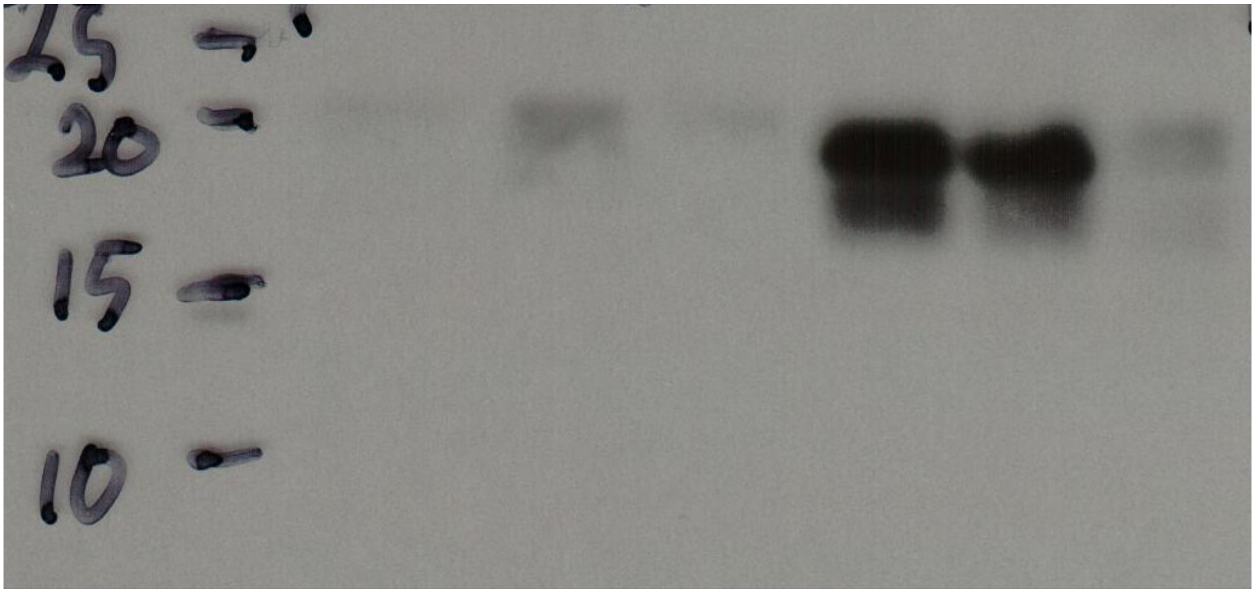

Fig S4g: Cl. Casp 3(17,19kDa) lanes 1-3 -- saline

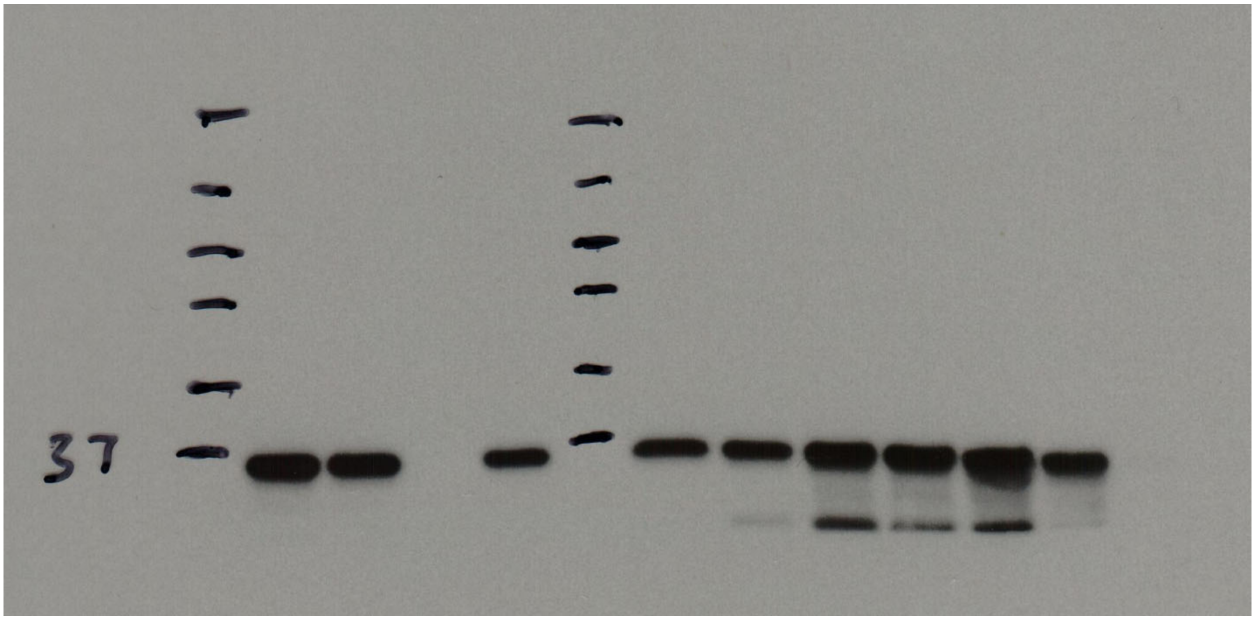

Fig S4g: GAPDH(37kDa) right above this box lanes 1-3 -- saline

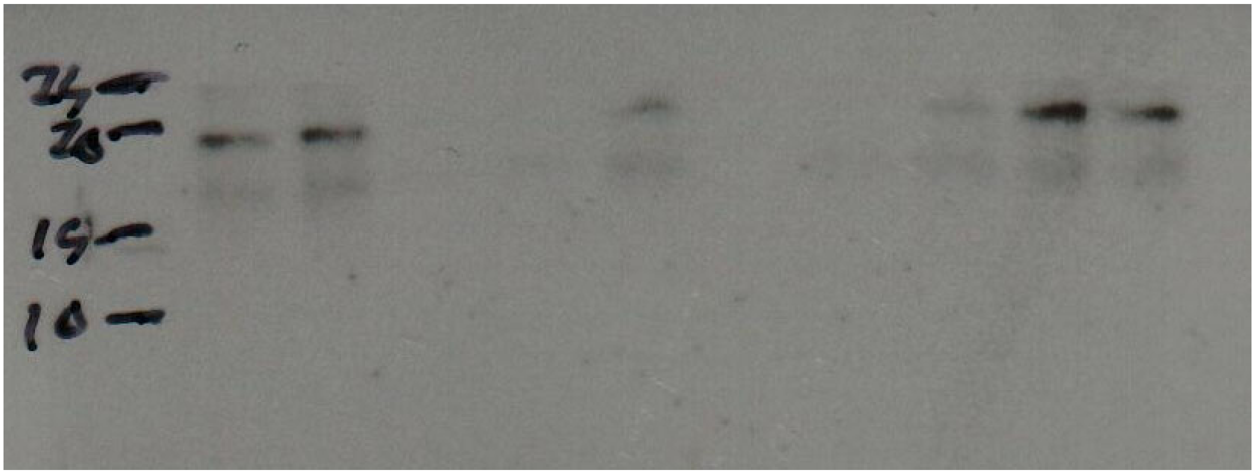

Fig S4g: Cl. Casp 3 (17,19 kDa) right above this box lanes 9-11 --OVA

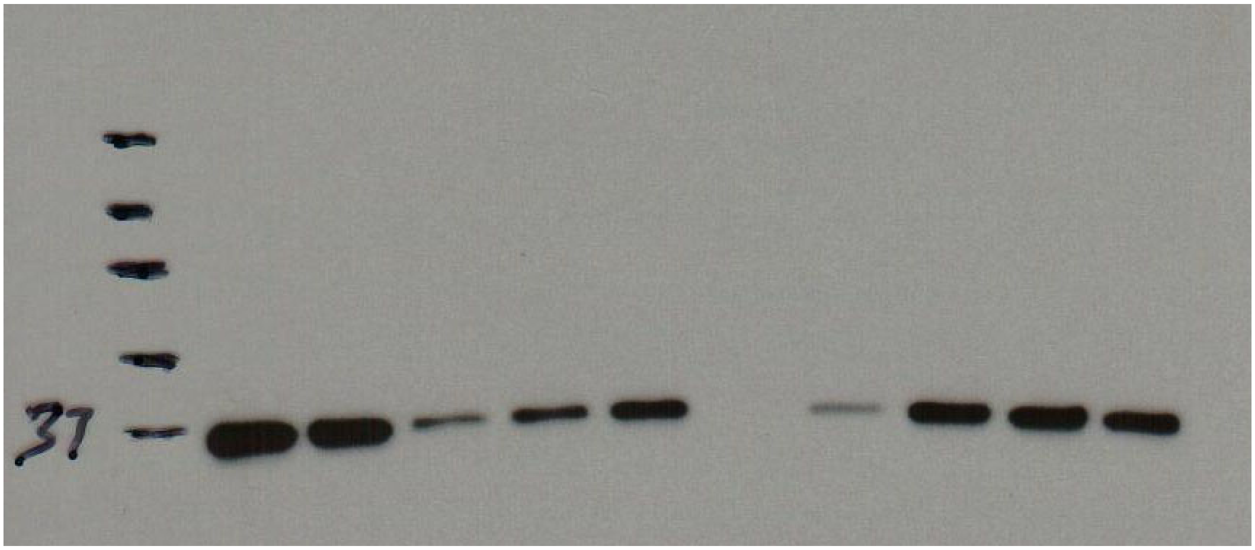

Fig S4g: GAPDH (37kDa) right above this box lanes 9-11 --OVA

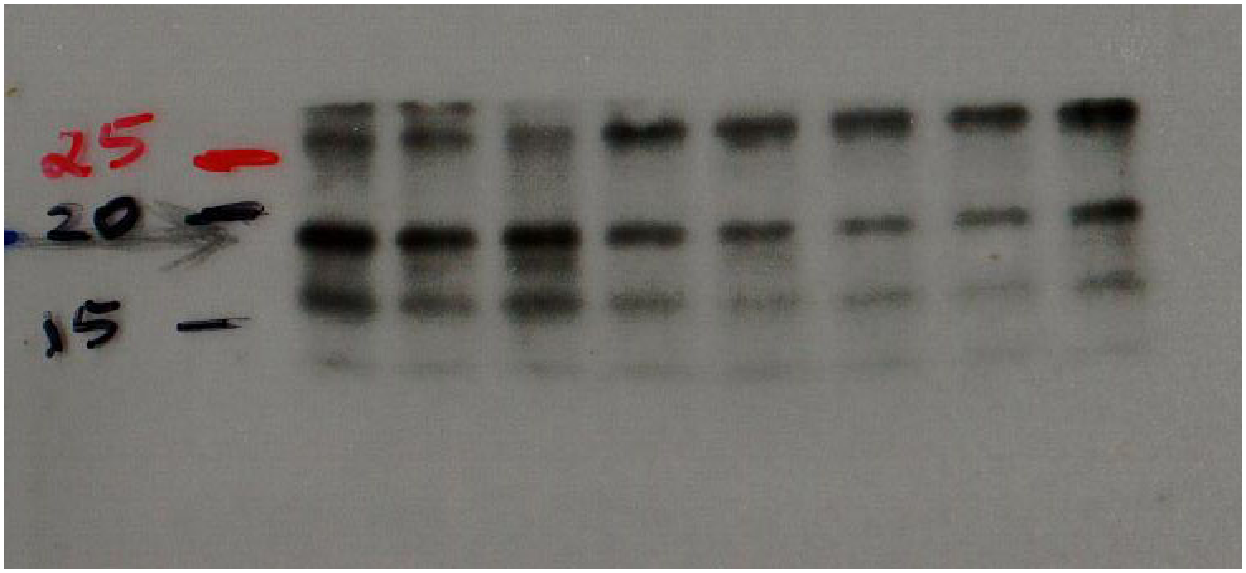

Fig S4g: Cl. Casp 3 (17, 19kDa) right above this box lanes 5-7 – reconst OVA

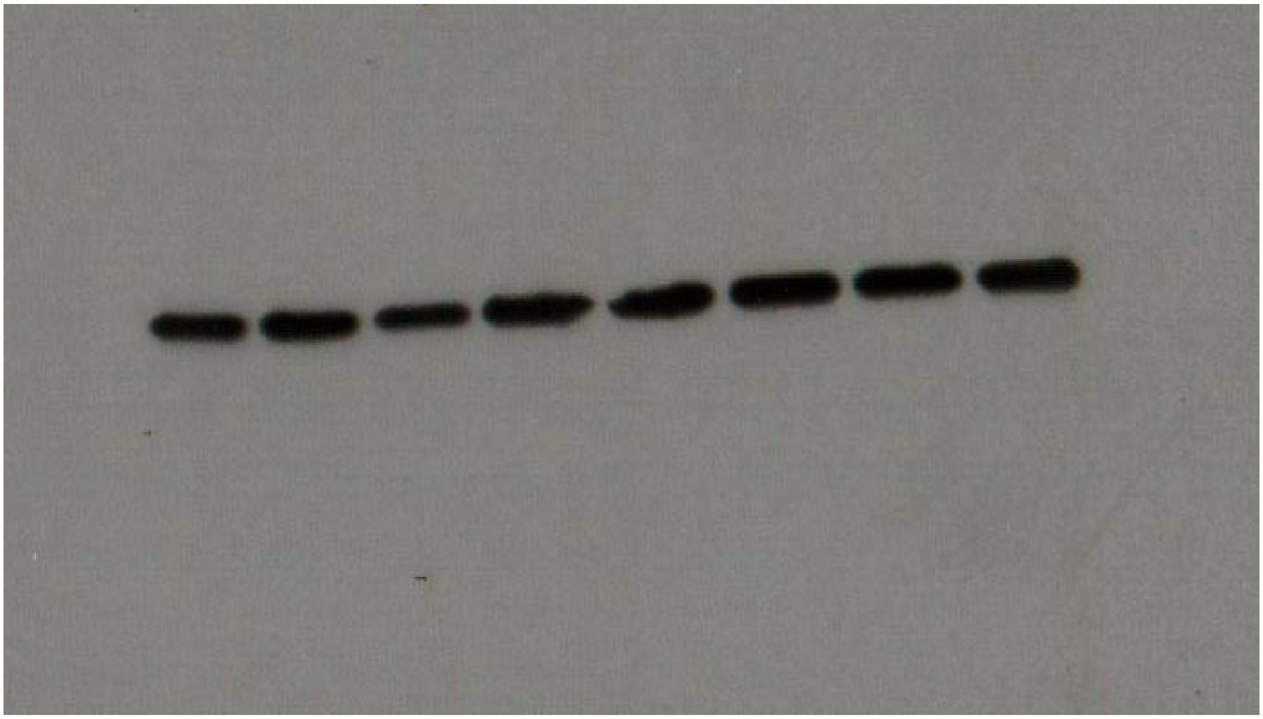

Fig S4g: GAPDH (37kDa) right above this box
lanes 4-6 – reconst OVA

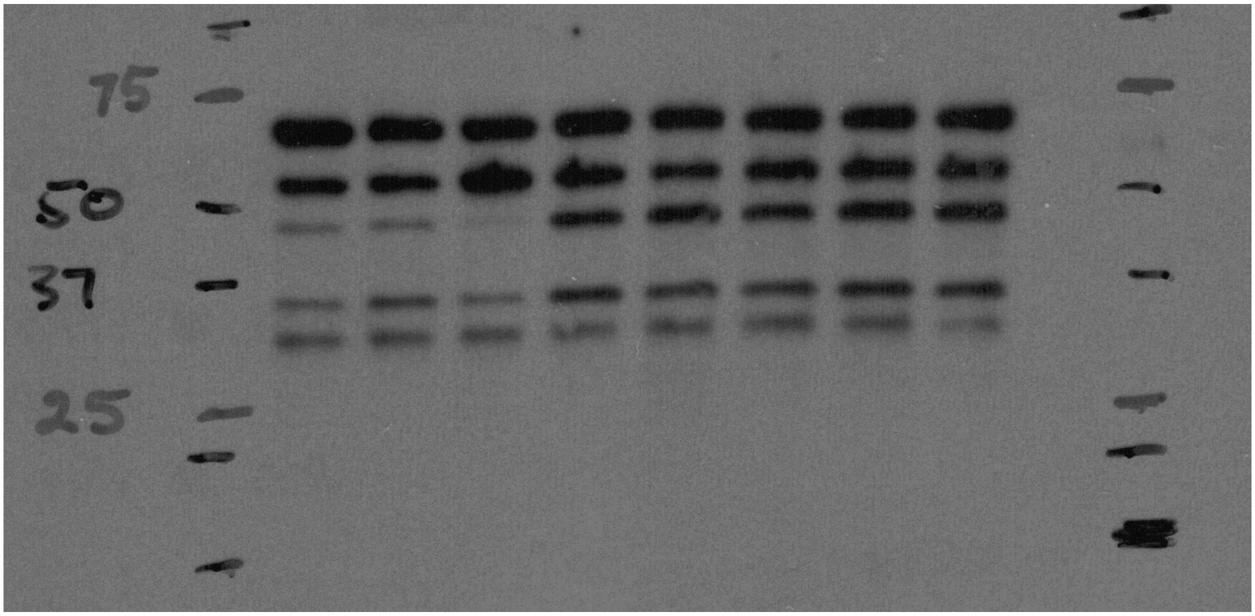

Fig S4i: Chop (27kDa) right above this box: saline (lanes 2-4) and reconst OVA (lanes 5-9)

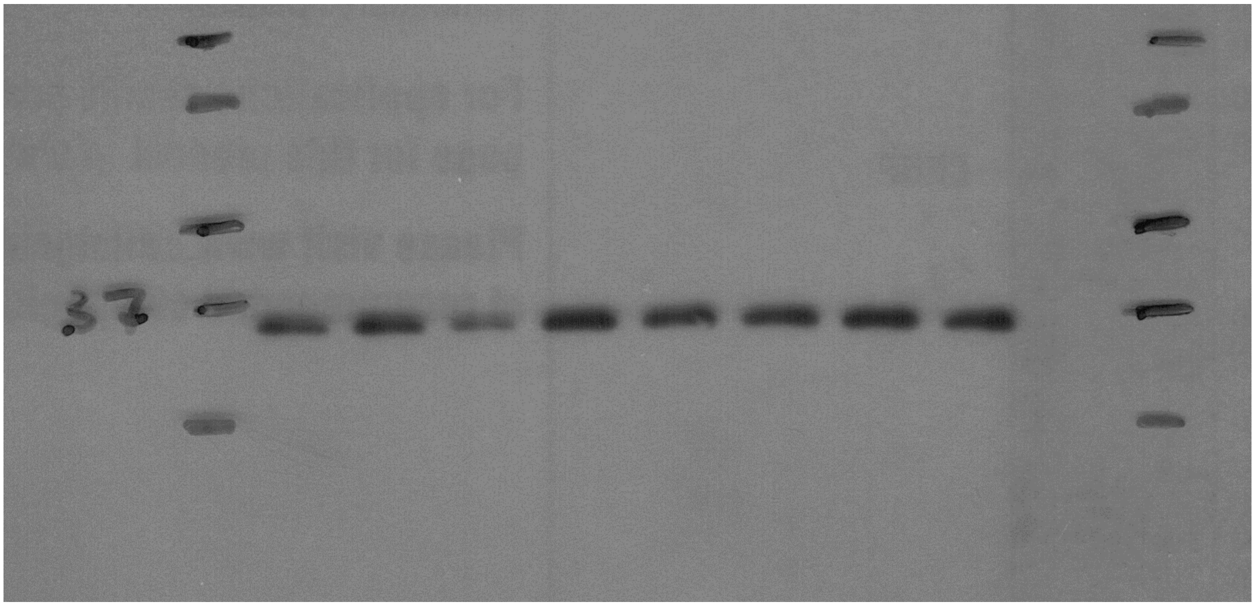

Fig S4i: GAPDH (37kDa) right above this box: saline (lanes 2-4) and reconst OVA (lanes 5-9)
